# Ultrasound monitoring of corpus luteum morphological evolution and serum progesterone concentration in pregnant and non-pregnant dogs: A prospective, observational study

**DOI:** 10.1016/j.vas.2025.100444

**Published:** 2025-03-20

**Authors:** Alessandra Paganotto, Camille Langlade, Samuel Buff, Émilie Rosset

**Affiliations:** Université de Lyon, VetAgro Sup, 1 avenue Bourgelat, 69280 Marcy l'Etoile, France

**Keywords:** Corpus luteum, Ultrasound, Pregnancy, Bitch, Progesterone, Follicle

## Abstract

The corpus luteum is the only structure producing progesterone during pregnancy in dogs. The aim of this study was to characterise morphological changes of corpora lutea in the bitch and assess their relationship with body weight, serum progesterone concentration, and multiple resorptions. We monitored 26 bitches weekly from ovulation confirmation to 35 days post-ovulation, measuring the corpora lutea diameter via ultrasound examination in combination with progesterone assays. The pregnancy rate was 80.7% (21/26), and all pregnancies were carried to term. Dogs were classified into small (5–15 kg), medium (16–39 kg), and large breeds (40–65 kg). Dog weight significantly influenced mean luteal diameter (*P* < 0.001), which ranged from a mean ± SD of 3.4 ± 0.5 mm for small dogs to 6.0 ± 0.7 mm for large dogs on the day of ovulation confirmation. From ovulation confirmation to peak, corpora lutea grew significantly (2.1 ± 1.2 mm; *P* < 0.001) and returned to their initial size by day 35. Surprisingly, one-third of maximum corpora lutea exceeded 1 cm before undergoing subsequent physiological diametric reduction. This growth in luteal diameter was positively correlated with serum progesterone concentration (*P* < 0.05). This study provides novel findings on canine corpus luteum characteristics, not previously described in literature, which could aid ovulation detection and differentiation between physiological and potentially pathological structures.

## Highlights

• Corpora lutea diameter and number vary with bitch size.
• Post-ovulation, dogs often develop cavitary corpora lutea >1 cm that physiologically reduce.
• There is a significant correlation between plasma progesterone and luteal diameter.
• Luteal diameter does not vary between pregnant and non-pregnant dogs.

## 1 Introduction

Progesterone is a crucial hormone for maintaining pregnancy in the bitch. It inhibits follicular development and ovulation during dioestrus (Senger 2012). The corpus luteum is the only structure producing progesterone during pregnancy in dogs. Ovariectomy at any stage of pregnancy will result in resorption or abortion within 48–56 hours (Sokolowski 1971; Tsutsui 1983). Although the corpus luteum autonomously produces progesterone, other hormones, such as prolactin, are known to have a luteotropic role in canines (Kowalewski 2014). While multiple studies were undertaken to observe corpora lutea evolution in the bitch during dioestrus (Marinelli et al. 2009; Stornelli et al. 2020; Nogueira Aires et al. 2022), they either conducted observations after ovariohysterectomy or in non-pregnant bitches. Ovariohysterectomy is not a valuable option for bitches that are destined to reproduce.

Ultrasonography, combined with serum progesterone, is a reliable method for detecting the onset of ovulation in dogs (Tsuchida et al. 2022). This approach also enables the observation of corpora lutea, which are fluid-filled during ovulation and subsequently characterised by gradual thickening of the antral wall and obliteration of the central anechoic cavity (England and Yeager 1993; Hayer et al. 1993). Ultrasound is thus a practical and non-invasive tool for monitoring corpus luteum during pregnancy without the need for ovariohysterectomy.

The diameter of the corpus luteum is positively correlated with progesterone production in cows and mares (Kayacik 2005; Pinaffi et al. 2015; Ishak et al. 2017). In women, the analysis of its development is useful in assessing pregnancy viability (Glock et al. 1995). The corpus luteum has only been studied to a limited extent in pregnant bitches, leaving uncertainties regarding the possible correlation of its size with progesterone production and its potential as an indicator of luteal deficiency.

In cattle, luteal deficiency during the embryonic phase is associated with a lower conception rate and early pregnancy loss. As a result, progesterone treatment is routinely used in low-fertility animals (López-Gatius and Garcia-Ispierto 2022). In mares, a decrease in plasma progesterone concentration during the early luteal phase results in increased early embryonic loss and delayed foetal growth after placentation (Wagner et al. 2023). In sows, the progesterone concentration at the time of pregnancy recognition is correlated with the number of live-born piglets, making it a predictive indicator for pregnancy outcome (Liu et al. 2020).

While luteal deficiency seems to be an uncommon condition of the bitch pregnancy (Hinderer et al. 2021), abortions due to low progesterone concentration before pregnancy term are reported (Görlinger et al. 2005; Günzel-Apel et al. 2006; Krachudel et al. 2013).

The aim of this study was to describe corpus luteum development throughout pregnancy and to examine the correlation between body weight, serum progesterone concentration, follicular size, resorption occurrence, and luteal size development. As we collected all information for non-pregnant bitches until day 21, we decided to also analyse their measurements to highlight possible differences between pregnant and non-pregnant bitches.

## 2 Material and methods

### 2.1 Animals and protocol

Animals were enrolled in the study from 2022 to 2023. The protocol was offered to all owners provided they agreed to commit to the weekly examination during the bitch's pregnancy. All bitches were in good health, correctly dewormed and vaccinated and had not received any treatment prior to enrolment. Blood samples were collected from each dog by cephalic venipuncture and then centrifuged at 5000 rpm for 5 minutes to obtain serum. Samples were collected at intervals of 1–3 days during proestrus and oestrus and continued until ovulation confirmation on Day 0. Ovulation was confirmed by ultrasound examination with the presence of ovarian effusion between 24 and 72 hours after observing progesterone concentration of 2–3 ng/mL, corresponding to the LH peak (Kutzler et al. 2003). Progesterone assays were performed using an Immulite 2000 apparatus (Siemens Healthcare GmbH, Erlangen, Germany) according to the method described by Fontbonne et al. (2021) under ISO 15189 certification. Reproduction was by natural mating or artificial insemination, depending on owner requirements. Blood samples were also collected on the day of insemination when performed at VetAgro Sup. From then on, blood samples were collected every 7±1 days from the day of ovulation until the end of gestation. Ovarian ultrasound was performed concurrently with each blood sample collection to measure follicle diameters before ovulation and corpus luteum diameters after ovulation. Gestation was confirmed by ultrasound on day 21±1 post-ovulation. Non-pregnant bitches followed the same examination schedule until monitoring was discontinued at this time.

The Canon Xario 100G ultrasound system was used to perform B-mode ultrasound examinations on standing dogs using a high-resolution 7.0 MHz curved array probe (15 mm). Dogs underwent abdominal hair clipping before being transferred to the examination room. The ovaries were examined transabdominally by a team of four veterinarians. All examinations were performed prior to obtaining progesterone concentration. The luteal tissue diameter was calculated as the average of its length and width and the mean luteal diameter was determined by averaging the diameter of all corpora lutea across both ovaries. Additionally, the maximum luteal diameter, representing the largest corpus luteum, was analysed as a faster and more practical clinical measurement with the potential to correlate with overall luteal function. Foetal vitality and foetal number were assessed consistently, and any incidence of embryonic resorptions was recorded.

### 2.3 Statistical analysis

The statistical analysis was conducted using the rstatix package on R Studio® software version 2024.04.0. The dogs were classified into small (5–15 kg), medium (16–39 kg), and large breeds (40–65 kg) based on their body weight. The maximum and mean luteal diameters were analysed as dependent variables in a repeated measures mixed design analysis of variance (ANOVA), with days post-ovulation as the repeated measure and the following subgroups as fixed factors: body weight groups, pregnant versus non-pregnant, and presence of multiple resorptions. Shapiro-Wilk tests of normality were also performed, and the conditions were met. *P*-values were adjusted for multiple comparisons using the Bonferroni correction method. Pearson's correlation coefficient was used to assess the correlation between continuous variables. Results were considered significant if *P* < 0.05. Data are presented as mean ± standard deviation (SD) unless otherwise stated.

## 3 Results

### 3.1 General results

The study included 26 bitches from different breeds, of which 21 were confirmed pregnant during follow-up. Among the pregnant bitches, 7 were small breeds, 7 medium breeds, and 7 large breeds, with a mean age of 3.3 ± 1.4 years and a mean body weight of 27.5 ± 18.9 kg (range: 6–65). The five non-pregnant bitches included three small breeds and two large breeds, with a mean age of 3.0 ± 1.0 years and a mean body weight of 25.4 ± 27.9 kg (range: 5–65). Further cohort details are available in **Supplementary Table S1**.

Ultrasound scans were used to assess luteal tissue, but this was found to be difficult after day 35 post-ovulation due to the effect of foetal mass on ovarian position. Luteal tissue assessment was therefore limited to the period up to day 35 post-ovulation. The maximum and mean luteal diameters across ovaries were strongly correlated (r = 0.95; *P* < 0.001, **Supplementary Table S2**). The maximum and mean follicular diameters were, on average, 5.1 ± 1.0 mm (range: 2.9–8.6) and 4.5 ± 0.9 mm at the last preovulatory examination, 2.6 ± 0.9 days before ovulation. A positive linear correlation was also observed between the mean follicle diameter at the last preovulatory examination and the mean luteal diameter after ovulation (r = 0.61; *P* = 0.007), and between the maximum follicular and luteal diameters (r = 0.66; *P* = 0.003).

### 3.2 Evolution of corpora lutea over time in pregnant dogs

**Fig. 1:**
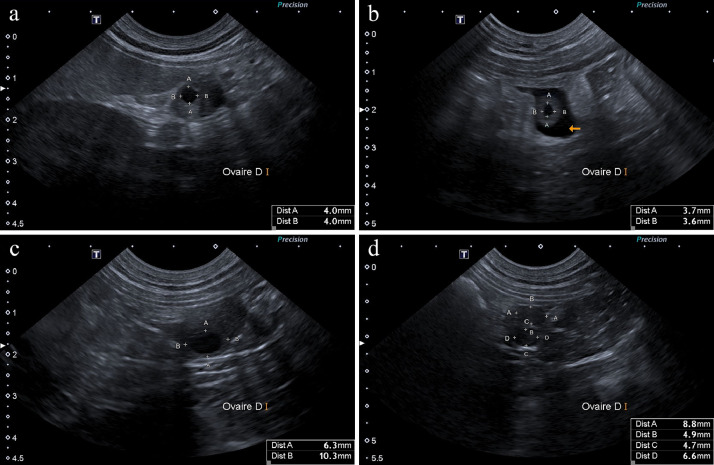
Ovarian structures evolution in the same pregnant bitch (ID 18) during oestrus and dioestrus. Anechoic follicles (a: Day -3) show a reduction of their anechoic cavity, associated to peri-ovarian effusion (arrow, b: Day 0), at ovulation. The forming corpus luteum enlarges its anechoic lumen and thickens its wall (c: Day 8). Its development during dioestrus is characterised by a progressive antral wall thickening and obliteration of the central anechoic lumen (d: Day 21).

**Fig. 2:**
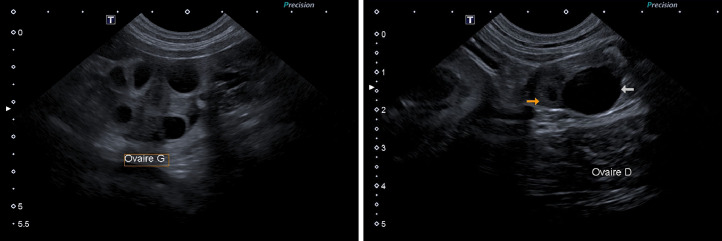
Comparison of corpora lutea within the same ovary at different stages of wall thickening, observed at day 2 post-ovulation (left, ID 14) and day 14 post-ovulation (right, ID 11). The orange arrow indicates a nearly complete tissular corpus luteum, and the white arrow indicates a larger (1.5 cm) corpus luteum still presenting a large anechoic lumen. While large corpora lutea could be suspected of being ovarian cysts, the ones observed in this study gradually lost their anechoic lumen to become completely tissular by day 21.

Preovulatory follicles had an anechoic appearance with circular shape and a relatively thin wall (Fig. 1a). Reduction of the anechoic central cavity and thickening of the antral wall was observed at the moment of ovulation, accompanied by peri-ovarian effusion and hyperechogenicity of the ovarian bursa (Fig. 1b). Following ovulation, corpora lutea were fluid-filled (Fig. 1c) and subsequently characterised by gradual thickening of the antral wall and obliteration of their anechoic lumen (Fig. 1d). Corpora lutea within the same ovary were often at heterogeneous stages of wall thickening (Fig. 2). By the 7th day post-ovulation, 62% (114/184) of corpora lutea had already lost their central lumen and appeared completely tissular, while 38% still appeared as fluid-filled cavities. By the 14th day post-ovulation, most corpora lutea (90%, 166/184) appeared entirely tissular. By the 21st day, all of them appeared tissular. No significant difference in progesterone peak concentration was found at any time point between bitches with fully tissular corpora lutea and those with heterogeneous stages of wall thickening.

**Fig. 3:**
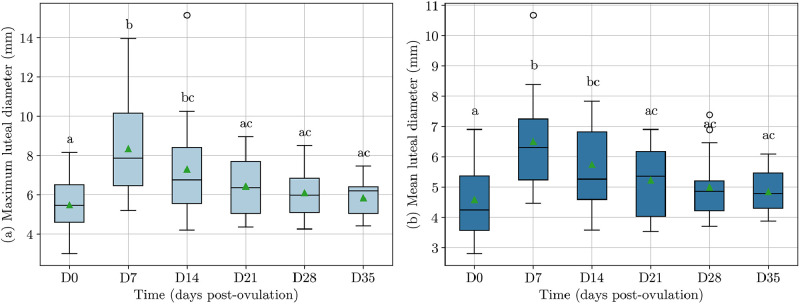
Boxplots showing maximum (a) and mean (b) luteal diameter (mm) evolution across days post-ovulation for 21 pregnant dogs. The box plot includes the median (line inside the box), interquartile range (box), outliers (dots), and mean (markers). Different superscript letters indicate statistical difference (P < 0.05) between days, as determined by an analysis of variance followed by Bonferroni's multiple comparison test.

Days post-ovulation significantly influenced both mean (*P* = 0.001) and maximum luteal diameters (*P* < 0.001), with the greatest difference observed between day 0 and day 7 (*P* < 0.001) (Fig. 3). On ovulation confirmation (Day 0), the mean and maximum luteal diameters were 4.6 ± 1.2 mm and 5.4 ± 1.4 mm (**Supplementary Table S2**). After ovulation, there was an increase in corpus luteum diameter, with a mean duration of increase of 8.5 ± 3.9 days. Out of the 21 dogs, 17 (81%) reached peak mean luteal diameter on day 7 ± 1 day and 3 (14%) on day 14 ± 1. The mean luteal diameter increased by an average of 2.1 ± 1.2 mm from ovulation confirmation to its peak, and the maximum by 3.4 ± 2.2 mm.

After ovulation, eight out of 21 pregnant dogs (38%) developed a corpus luteum larger than 1 cm, at an average of 1.2 ± 0.2 cm (range: 1.0–1.5 cm). They occurred in small (3/8), medium (2/8) and large breeds (3/8). These pregnancies lasted 62 ± 1 days, and 50% (4/8) of them had partial resorptions.

### 3.2 Range of corpora lutea diameter and number by body weight

**Fig. 4:**
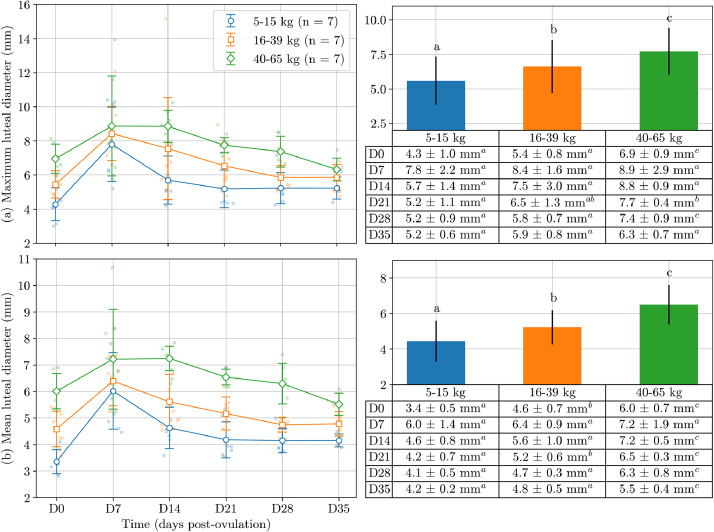
Scatter and bar plot of maximum (a) and mean (b) luteal diameters in 21 pregnant bitches measured weekly from day 0 to day 35 post-ovulation. Bitches are categorised into three weight groups, 5–15 kg (n = 7), 16–39 kg (n = 7), and 40–65 kg (n = 7). The bars represent the mean ± standard deviation across all days and the tables present the values for days 0, 7, 14, 21, 28, and 35 post-ovulation. Different superscript letters indicate statistical difference (*P* < 0.05) between body weight groups, as determined by an analysis of variance followed by Bonferroni's multiple comparison test.

Body weight groups showed significantly different mean (*P* < 0.001) and maximum luteal diameters (*P* = 0.001) (Fig. 4). There was a significant positive correlation between body weight and both mean (r = 0.72; *P* < 0.001) and maximum luteal diameter (r = 0.85; *P* < 0.001). Small dogs had significantly lower mean luteal diameter than large breeds. The effect of body weight on mean and maximum luteal diameter was significant across most days post-ovulation, notably on days 0, 14, 21, and 28 post-ovulation (*P* < 0.001).

**Fig. 5:**
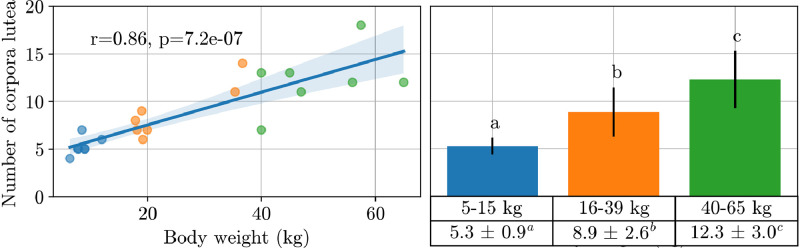
Left: Scatter plot showing the relationship between number of corpora lutea and body weight in 21 pregnant bitches. The regression line represents the linear fit, with the shaded area indicating the 95% confidence interval (Pearson's r = 0.86). Right: Bar plot of the number of corpora lutea categorised into three weight groups, 5–15 kg (n = 7), 16–39 kg (n = 7), and 40–65 kg (n = 7). Bars represent the mean ± standard deviation. Different superscript letters indicate statistical difference (*P* < 0.05) between weight groups, as determined by an analysis of variance.

The number of corpora lutea was highly correlated with body weight (r = 0.86; *P* < 0.001), with small, medium, and large dogs averaging 5.3 ± 0.9 (n = 7), 8.9 ± 2.6 (n = 7), and 12.3 ± 3.0 (n = 7) respectively (Fig. 5). There was a strong correlation between the number of corpora lutea and both mean (r = 0.65; *P* = 0.001) and maximum (r = 0.67; *P* < 0.001) luteal diameters, with fewer corpora lutea observed in cases of smaller corpora luteal diameters. This correlation was significant on most days post-ovulation, except day 7, and was highest on day 0 (r = 0.73; *P* < 0.001, and r = 0.75; *P* < 0.001, respectively).

### 3.3 Correlation between luteal diameter and progesterone concentration in pregnant dogs

**Fig. 6:**
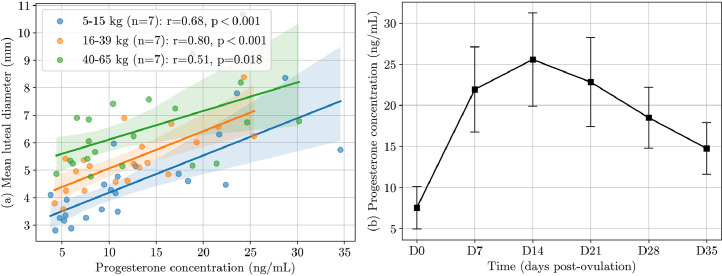
(a) Scatter plot, Pearson correlation coefficient, and P-value for progesterone concentration (ng/mL) versus mean luteal diameter in 21 pregnant bitches, among three body weight groups: 5–15 kg, 16–39 kg, 40–65 kg. Each point corresponds to an ovarian ultrasound performed concurrently with a blood sample between day 0 and 8 post-ovulation, and the continuous line with shaded regions represents the best-linear fit with 95% confidence intervals (CI). (b) Progesterone concentration (ng/mL) across days post-ovulation for 21 pregnant dogs, with mean values and standard deviations.

Both postovulatory progesterone concentration and luteal diameter showed an initial rapid increase after ovulation, followed by a gradual decrease (Fig. 3, Fig. 6). Variations in progesterone concentration between days 0 to 35 post-ovulation paralleled those observed in luteal diameter, with a moderate correlation (r = 0.30; *P* < 0.001). Their correlation was highest during the first week post-ovulation (r = 0.62; *P* < 0.001; Fig. 6). Progesterone concentration at day 7 was correlated with the luteal diameter increase in the first week post-ovulation (r = 0.50, *P* = 0.027). However, progesterone concentration peaked later than luteal diameter, at an average of 15.3 ± 6.3 days post-ovulation (range: 7–28). The mean peak progesterone concentration was 26.1 ± 5.6 ng/mL (range: 17.1–34.6). No significant correlation was observed between progesterone concentration and the number of corpora lutea at any time point post-ovulation.

### 3.4 Correlation between luteal diameter, resorption and litter size

Partial resorptions were observed in 48% (10/21) of the dogs, with an average observation time of 30 ± 3 days post-ovulation (range: 27–35). Among these cases, 30% (3/10) were multiple resorptions and 70% (7/10) were single resorptions. There were no significant differences in luteal diameter between bitches with multiple resorptions and those with one or none (*P* = 0.86).

Of the 21 pregnant bitches included in the study, ten whelped naturally, seven underwent elective caesarean sections and four required emergency caesarean sections, two for primary uterine atony and two for secondary uterine atony. Excluding elective caesarean sections, the mean gestation length was 61.8 ± 1.2 days (14/21). The mean litter size was 6.6 ± 3.8, with a total of 134 puppies. Number of corpora lutea was highly correlated with the litter size (n = 21; r = 0.95; *P* < 0.001). The mean ratio between the number of foetuses and corpora lutea was 0.75 ± 0.28.

### 3.5 Non-pregnant versus pregnant dogs

When comparing pregnant (n = 21) and non-pregnant bitches (n = 5), no significant difference was observed for luteal diameter over time (*P* = 0.12, Table 1) nor progesterone concentration (*P* = 0.34). Both showed the same initial increase in luteal diameter after ovulation, followed by a gradual reduction.

**Table 1:** Mean and maximum luteal diameter (mm) across days post-ovulation for 21 pregnant dogs and 5 non-pregnant dogs, with mean values and standard deviations.

	D0		D7		D14		D21	
	Mean	Max	Mean	Max	Mean	Max	Mean	Max
Pregnant (n = 21)	4.6±1.2	5.4±1.4	6.5±1.5	8.3±2.3	5.7±1.3	7.3±2.4	5.2±1.1	6.4±1.4
Non-pregnant (n = 5)	4.2±1.1	5.3±1.7	7.0±1.7	9.2±2.7	5.0±1.2	6.2±1.5	4.3±1.0	5.2±1.2

## 4 Discussion

Our study showed a significant increase in luteal diameter during the first week post-ovulation. To our knowledge, this observation was not previously described in the literature for the canine species. It is consistent with findings in cattle reported by Gómez-Seco et al. (2017). Luteal growth preceded the rise in progesterone concentration, with luteal diameter peaking before the progesterone surge. The progesterone concentration in our study, which peaked on average at 15.3 ± 6.3 days of diestrus, is consistent with previous literature (Concannon 2011; Hinderer et al. 2021). Previous studies in cows (Kayacik 2005; Mann 2009), goats (Arashiro et al. 2010), and mares (Ishak et al. 2017) have demonstrated a correlation between luteal size and progesterone concentration. A correlation has been observed in dogs between luteal weight and progesterone concentration during dioestrus (Marinelli et al. 2009), as well as between luteal volume and progesterone concentration in early dioestrus (Stornelli et al. 2020). Our study also found a correlation between luteal diameter and progesterone concentration during the first week after ovulation. The shorter time frame in comparison to the existing literature may be attributed to the wide range of ovulation rates and luteal tissue weights in dogs. The correlation we found between progesterone concentration and luteal size opens an interesting prospect for monitoring bitches at risk of luteal insufficiency. Luteal insufficiency is described as a condition where there is an early decline in the concentration of progesterone in the bloodstream, leading to pregnancy loss during the luteal phase. In cattle, luteal cell growth and vascularisation correlate with progesterone secretion in early pregnancy (Gómez-Seco et al., 2017). Reduced corpus luteum growth from ovulation to peak diameter may indicate potential deficiencies in luteal tissue quality and vascularisation. Thus, ultrasound monitoring of the corpora lutea could be associated with progesterone assay during early gestation to monitor any abnormal evolution of the corpora lutea.

As reported in literature (Marinelli et al. 2009), we found strong correlations between corpora lutea number and both the bitch's body weight and litter size. Additionally, higher corpora lutea number was associated with larger mean and maximum luteal diameters. These results highlight the potential of ovarian ultrasound for fertility evaluation and prediction even after ovulation. Moreover, the strong correlation between mean and maximum luteal diameters suggests that measuring the maximum luteal diameter may be preferable due to its faster process.

Our study found a strong positive correlation between body weight and luteal diameter at ovulation and dioestrus, which is consistent with Marinelli et al.’s (2009) observation, but in contrast to Stornelli et al.’s (2020) findings. The correlations we observed between luteal number and body weight provide further support for these associations. Stornelli et al. (2020) may have obtained different results due to their narrower weight range (2.5–25 kg) and reliance on single measurements obtained by surgical ovariohysterectomy. Our results, contrary to Stornelli et al. (2020), did not find a correlation between the number of corpora lutea and progesterone concentration. Since we observed a correlation between the number of corpora lutea and the bitch body weight, our finding is consistent with the absence of correlation between the bitch weight and progesterone concentration during either estrus (Hollinshead et Hanlon, 2019) and diestrus (Hindered et al. 2021).

Our study showed a correlation between the diameter of preovulatory follicles and that of corpora lutea at the time of ovulation, consistent with prior observations in cattle (Pinaffi et al. 2015).

The follicles’ diameter should remain under 8 mm (Arlt and Haimerl 2016), as observed in our study. Ovarian structures exceeding 1 cm during oestrus warrant consideration as potential ovarian cysts. However, this criterion may not apply to corpora lutea. We noted multiple corpora lutea surpassing 1 cm during the week following ovulation, all of which regressed during dioestrus without adversely affecting gestation. Should the suspicion of a cyst arise, we recommend a follow-up examination two weeks post-ovulation, as corpora lutea typically undergo physiological diametric reduction during this stage of dioestrus.

In cows, corpora lutea can present themself as either homogeneous or cavitary, and the functional difference in progesterone productions has been studied with contrasting results (Perez-Marin 2009; Jaśkowski et al. 2022). In our study, a faster loss of anechoic lumen of the corpora lutea did not appear to influence progesterone concentration. This may be due to variations in the development of individual corpora lutea within the same ovary, resulting in different degrees of maturation occurring simultaneously—a phenomenon rarely observed in monotocous species. Corpora lutea began to lose their anechoic lumen around day 7 post-ovulation, reaching a near-complete transition to a homogeneous tissular appearance by day 21. The presence of large anechoic lumen in the corpora lutea following ovulation was not indicative of pathology. Resembling preovulatory follicles, corpora lutea with large anechoic lumen present in the week after ovulation may complicate accurate ovulation detection without regular ultrasound monitoring during heat. However, the presence of both tissular and fluid-filled structures in an ultrasound scan is in favour of an ovulation that has already occurred.

While the number of observed non-pregnant bitches in our study was limited, we did not observe any variation in corpora lutea evolution in either morphology or diameter increase between non-pregnant and pregnant bitches. This finding aligns with the notion that progesterone concentration does not differ between early gestation and non-pregnant diestrus in the bitch (Luz et al. 2006; Hinderer et al, 2021).

The bitch's unique ovarian physiology presents challenges when monitoring periovulatory ovarian activity. Luteinization of follicles occurs before ovulation and follicles do not collapse after ovulation, making it difficult to distinguish between preovulatory follicles and corpora lutea (Bergeron et al. 2013). Still, in our study, a close ultrasound examination after LH peak was detected allowed for detection of the periovulatory thickening of the follicular wall, the disappearance of the anechoic antrum at ovulation and the formation of thickened wall corpora lutea. It was not possible however to obtain precise measurement after 35 days post ovulation, limiting our observation to the first month of dioestrus. However, since luteal diameter and progesterone productions are correlated in our study only in the first weeks after ovulation, this seems to have a small impact on our results.

In our study, resorptions occurred in 48% of pregnancies. While isolated asymptomatic resorptions can affect up to 48.3% of pregnancies (Lascialfari et al. 2023), they can represent an important loss for the breeder. Observation of multiple resorptions showed no correlation with corpora lutea diameter.

The weekly measurement schedule may have affected the accuracy of our results by potentially missing changes. To address these limitations, future research could increase the frequency of examinations and use advanced ultrasound systems to improve precision.

## 5 Conclusions

To our knowledge, this study is the first to use ultrasound to analyse how ovarian structures develop during oestrus and subsequent pregnancy in the bitch, and their relationship with serum progesterone concentration. The correlation found between progesterone concentration and luteal diameter during early pregnancy opens a novel research area on early identification of bitches at risk of progesterone deficiency. Future studies should assess the diagnostic potential of corpus luteum measurement in identifying luteal insufficiency. Our study also provides a detailed description of the corpora lutea diameter normal range and appearance throughout early canine pregnancy, which could contribute to improving ovulation detection, pregnancy monitoring, and differentiation of physiological from possibly pathological structures.

## Ethical statement

The research was conducted at VetAgro Sup and approved by the Research Ethics Committee of the University of Lyon (approval number n 2251).

## Funding

The authors declare that no funds, grants, or other support were received during the preparation of this manuscript.

## Data availability

The data that support the findings of this study are available from the corresponding author upon reasonable request.

## CRediT authorship contribution statement

**Alessandra Paganotto:** Writing – original draft, Investigation, Formal analysis, Data curation, Conceptualization. **Camille Langlade:** Writing – review & editing, Investigation, Conceptualization. **Samuel Buff:** Writing – review & editing, Investigation, Conceptualization. **Émilie Rosset:** Writing – review & editing, Investigation, Conceptualization.

## Declaration of competing interest

The authors have no relevant financial or non-financial interests to disclose.

## Appendix Supplementary materials

Image, application 1

Image, application 2
