# Supplementary material for: Ultrasound monitoring of corpus luteum morphological evolution and serum progesterone concentration in pregnant and non-pregnant dogs: A prospective, observational study
Source: Vet Anim Sci. 2025 Mar 20;28:100444. doi: 10.1016/j.vas.2025.100444 (PMC11986551; doi:10.1016/j.vas.2025.100444)
Supplement: Supplementary file 1 [file mmc1.pdf]

Supplementary table S1. Individual characteristics of dogs included in this study. Pregnancy-related fields are marked as 'Not Applicable' (NA) for non-pregnant dogs. EMCS: Emergency C-section, CS: Elective C-section, NW: Natural Whelping, OVH: Ovariohysterectomy.

Nr	Breed	Weight at first presentation		Primiparous	Age (y)	Nb corpora lutea	Nb embryos	Resorptions		Litter size (incl. stillborn)	Neonatal mortality		Whelping		
		Weight (kg)	Category					Nb	Day of observation		Nb deaths	Reason of death	Type	Day	Observations
01	Beagle	8,5	Small	Y	2	7	5	0		5	0		NW	61	
02	Beagle	7,9	Small	Y	2	5	4	1	35	4	1	One puppy died of cardiac malformation	CS	60	
03	Australian Sheperd	19,2	Medium	N	5	6	6	1	31	5	0		NW	60	
04	Dobermann	35,4	Medium	N	3	11	8	0		10	2	One puppy with hypospadias, euthanised, one puppy died of aspiration pneumonia	EMCS	63	
05	Dobermann	36,7	Medium	N	3	14	8	0		12	1	Stillbirth	NW	62	
06	Leonberg	57,5	Large	Y	4	18	>10	1	29	16	0		EMCS	60	
07	Beauce Sheperd	40	Large	N	7	6	5	0		5	0		NW	62	Mild mastitis
08	Beagle	7,8	Small	Y	2,5	5	4	0		5	1	One puppy crushed by the mother	NW	61	
09	Leonberg	56	Large	N	4	12	6	0		6	0		CS (Aglepristone injection)	61	
10	Australian Sheperd	19	Medium	Y	2	9	6	0		8	1	One puppy stillbirth	NW	61	
11	Alpine Dachsbracke	17,9	Medium	Y	4	8	5	0		7	0		EMCS	63	Severe mastitis
12	Saint Bernard	45	Large	Y	3	13	4	2	33	2	0		CS (Aglepristone injection)	63	OVH performed 24h after elective c-section
13	Newfoundland dog	47	Large	Y	3	11	8	2	28	9	3	Three puppies died of possible sepsis (no necropsies available)	CS (Aglepristone injection)	60	
14	Great Dane	65	Large	N	5	12	5	3	32	2	1	One puppy died of hypoxia (no necroscopy available)	CS (Aglepristone injection)	60	
15	Dobermann	40	Large	N	2	13	8	1	27	12	1	One stillbirth	NW	62	
16	Boston Terrier	9	Small	N	3	5	2	1	27	1	1	One puppy with cleft palat, euthanised	CS (Aglepristone injection)	62	

17	Boston Terrier	9	Small	N	2	5	3	0		5	0		NW	61	
18	Bull Terrier	18,2	Medium	Y	2	7	7	0		7	0		NW	63	
19	Boston Terrier	6,4	Small	N	2	4	4	1	27	4	0		CS (Aglepristone injection)	63	
20	English Setter	20	Medium	Y	6	7	4	0		6	1	One puppy died (half weight of the others)	EMCS	64	
21	Lagotto Romagnolo	12	Small	Y	3	6	3	1	25	2	0		NW	62	
22	Boston Terrier	5,5	Small	N	2	4	NA	NA		NA	NA		NA	NA	
23	Beauce Sheperd	45	Large	Y	4	10	NA	NA		NA	NA		NW	NA	
24	Pomeranian Spitz	5	Small	Y	3	4	NA	NA		NA	NA		NA	NA	
25	Great Dane	65	Large	N	4	13	NA	NA		NA	NA		NA	NA	
26	Boston Terrier	6,5	Small	Y	2	4	NA	NA		NA	NA		NA	NA	
