# Supplementary material for: Ultrasound monitoring of corpus luteum morphological evolution and serum progesterone concentration in pregnant and non-pregnant dogs: A prospective, observational study
Source: Vet Anim Sci. 2025 Mar 20;28:100444. doi: 10.1016/j.vas.2025.100444 (PMC11986551; doi:10.1016/j.vas.2025.100444)
Supplement: Supplementary file 2 [file mmc2.pdf]

Correlation between mean and maximum diameters for 21 pregnant dogs

Δ y - x = +14.2 ± 10.3% (r = 0.95, P < 0.001)

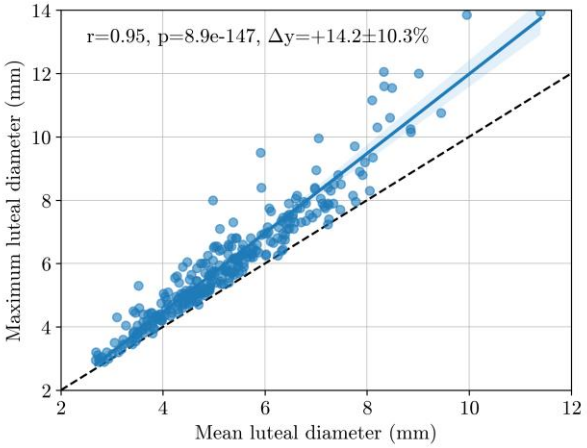

Mean luteal diameter range (mm) for 21 pregnant dogs

Day	Mean	Mean - SD	Mean + SD	Range (min,max)
0	4.6	3.4	5.8	(2.8 - 6.9)
7	6.5	5.0	8.0	(4.5 - 10.7)
14	5.7	4.4	7.1	(3.6 - 7.8)
21	5.2	4.1	6.3	(3.6 - 6.9)
28	5.0	4.0	6.0	(3.7 - 7.4)
35	4.8	4.2	5.5	(3.9 - 6.1)
All	5.3	4.0	6.7	(2.8 - 10.7)

Maximum luteal diameter range (mm) for 21 pregnant dogs

Day	Mean	Mean - SD	Mean + SD	Range (min,max)
0	5.4	4.1	6.8	(3.0 - 8.2)
7	8.3	6.1	10.6	(5.2 - 14.0
14	7.3	4.9	9.7	(4.2 - 15.1)
21	6.4	5.0	7.9	(4.3 - 9.0)
28	6.1	4.9	7.3	(4.3 - 8.5)
35	5.8	5.0	6.7	(4.4 - 7.5)
All	6.6	4.6	8.6	(3.0 - 15.1)
